# Global, regional, and national burden of gallbladder and biliary tract cancer from 1990 to 2021 and projections to 2050: a systematic analysis integrating the Global Burden of Disease 2021 and Mendelian randomization

**DOI:** 10.1097/JS9.0000000000005043

**Published:** 2026-05-11

**Authors:** Zhaowei Sun, Junzhe Su, Qinlei Wang, Haoran Li, Jingyun Guo, Jingru Zhang, Zelin Wang, Yanfeng Liu, Bingyuan Zhang, Yujie Feng

**Affiliations:** Department of Hepatobiliary and Pancreatic Surgery, The Affiliated Hospital of Qingdao University, Qingdao City, Shandong Province, China

**Keywords:** gallbladder and biliary tract cancer, global burden of disease, high BMI, Mendelian randomization, socio-demographic index

## Abstract

**Background:**

Gallbladder and biliary tract cancer (GBTC) imposes a growing global public health burden, with significant socioeconomic disparities. We assessed trends from 1990 to 2021, quantified inequalities, projected future burden, and evaluated the causal role of high body mass index (BMI) using Mendelian randomization (MR).

**Methods:**

Using Global Burden of Disease (GBD) 2021 data, we analyzed global/regional/national incidence, mortality, and disability-adjusted life years (DALYs) for GBTC. We decomposed drivers of burden changes, quantified cross-country inequalities with slope index of inequality, concentration index, projected trends to 2050 via Bayesian age–period–cohort modeling, and conducted two-sample MR to test BMI-GBTC causality.

**Results:**

Global GBTC prevalent cases increased by 142% (1990–2021), while age-standardized rates (ASRs) rose modestly (incidence EAPC = 0.97; mortality EAPC = 0.47). Burden was highest in high-socio-demographic index (SDI) regions, but low-SDI regions exhibited rising mortality and DALYs. Decomposition identified population growth as the primary burden driver across all SDI strata. Inequality analysis revealed persistent yet narrowing disparities. Projections indicated declining ASRs but rising absolute cases (+30% by 2050). MR confirmed a causal effect of elevated BMI on GBTC risk (OR = 1.0005 per unit BMI; 95% CI: 1.0000–1.0010; *P* = 0.043).

**Conclusions:**

GBTC burden growth is predominantly fueled by demographic forces, with worsening trends in resource-limited regions. The causal link between high BMI and GBTC underscores the need for targeted obesity interventions. Equitable prevention strategies and improved healthcare access are critical to mitigate disparities, particularly in low-SDI settings.

## Introduction

Gallbladder and biliary tract cancer (GBTC) has a low global incidence but extremely high lethality^[^1,2^]^. Surgical resection remains the only potentially curative modality^[^3^]^; however, due to the absence of obvious early symptoms, GBTC is often diagnosed at an advanced stage^[^4,5^]^. The median survival of patients with unresectable biliary tract cancer is only 3–6 months^[^6^]^. In 2021, GBTC ranked sixth in both incidence and mortality among global gastrointestinal malignancies, with 216 770 new cases and 171 960 deaths^[^7^]^. The high mortality and poor prognosis of GBTC underscore the necessity of effective therapeutic strategies, and the global burden of GBTC has posed a significant public health challenge for years.

HIGHLIGHTS

- The global burden of gallbladder and biliary tract cancer (GBTC) escalated markedly from 1990 to 2021.
- Burden in low-socio-demographic index (SDI) regions worsened persistently, with rising age-standardized mortality rate DALYs rates, reflecting inadequate healthcare and late diagnosis.
- Population growth was the dominant contributor to increased GBTC burden globally, outweighing aging and epidemiological changes across all SDI regions.
- Mendelian randomization confirmed a causal link: each 1-unit BMI increase elevated GBTC risk (OR = 1.0005, *P* = 0.043).
- By 2050, age-standardized rates are projected to decline, but absolute cases may rise 30% due to population growth/aging, with low-SDI regions facing exacerbated burden.

The epidemiology of GBTC is influenced by multiple factors including geographical location, gender, and age^[^8^]^. Multiple factors contribute to these disparities, including differences in dietary habits, obesity prevalence, and socioeconomic development levels^[^9^]^. In recent years, growing evidence has confirmed the association between rising prevalence of high body mass index (BMI) and multiple cancers, including GBTC^[^10,11^]^. As a major global health challenge, high BMI is thought to be closely linked to chronic inflammation, insulin resistance, abnormal energy supply to tumor cells, and altered hormonal regulation, contributing to cancer initiation and progression^[^12,13^]^. Recent epidemiological studies have shown a significant increase in cancer attributable to high global BMI, with cancer-related deaths and disability-adjusted life years (DALYs) increasing by 35% and 34%, respectively, between 2010 and 2019^[^14^]^. A clear relationship has been established between high BMI as a major risk factor and GBTC^[^15^]^. Quantifying the global burden of GBTC, especially high-BMI-attributable cases, is essential for developing evidence-based prevention policies. Geographic variations in GBTC burden data inform resource allocation and targeted prevention strategies by health authorities and researchers^[^16^]^.

In this study, we conducted a comprehensive assessment of Global Burden of Disease (GBD) data at global, regional, and national levels from 1990 to 2021, comparatively analyzing incidence, mortality, and DALYs of GBTC. By examining temporal trends in disease burden and projecting changes up to 2050, we aimed to inform cancer control and prevention strategies. This research also explored socioeconomic factors by investigating the socio-demographic index (SDI), age-specific differences, gender inequalities, and regional disparities to evaluate their impact on health outcomes. Furthermore, focusing on high BMI as a risk factor, we estimated the proportion of deaths and DALYs attributable to high BMI and performed two-sample Mendelian randomization (MR) analysis to validate the causal association between high BMI and GBTC. The findings of this analysis will provide critical insights for public health policies and initiatives aimed at mitigating the GBTC burden associated with high BMI.

Artificial intelligence (GPT-4) was used for language modification, grammar proofreading, translation, and reference formatting checking of this paper. The authors reviewed and edited the AI-generated content and take full responsibility for the truthfulness and accuracy of the content of this article. The use of artificial intelligence (AI) tools in the study complies with TITAN Guide 2025^[^17^]^.

## Methods

### Study population and data collection

This study utilized data from the Global Burden of Diseases, Injuries, and Risk Factors Study (GBD) 2021, conducted by the Institute for Health Metrics and Evaluation (IHME). This comprehensive systematic assessment quantifies the burden of 371 diseases and injuries and 88 risk factors across 204 countries and territories, 28 GBD super-regions, and 54 GBD regions from 1990 to 2021.

Specifically, we extracted age-standardized rates (ASRs) per 100 000 population and absolute counts for DALYs attributable to high BMI for GBTC. Data retrieval for 204 countries and 21 regions (1990–2021) employed the Global Health Data Exchange query tool, selecting “High Body Mass Index” as the risk factor and “Gallbladder and biliary tract cancer” as the specific cause. All estimates include 95% uncertainty intervals (UI). SDI values for each country were concurrently obtained.

### Definitions

SDI is a composite indicator used to assess regional socioeconomic development, integrating national-level per capita income, average educational attainment, and total fertility rate, with scores ranging from 0 (least developed) to 1 (most developed). Developed by GBD researchers, this index specifically incorporates the total fertility rate under 25 years (TFU25), average educational attainment for populations aged 15 and above (EDU15 +), and lag-distributed per capita income (LDI) to evaluate country-level development. In the *2021 Global Environment and Development Report*, SDI values were scaled by multiplying by 100 to a range of 0–100, where higher scores denote better socioeconomic conditions. Based on calculated SDI values, 204 countries and regions are categorized into five quintiles – high, high-middle, middle, low-middle, and low-SDI regions – to facilitate comparative analysis of socioeconomic disparities and their impact on health outcomes. High BMI was defined as BMI ≥ 25 kg/m2 for adults (aged 20 + years) and using thresholds from the International Obesity Task Force standards for children (aged <20 years).

### Statistical analysis

Primary outcome measures comprised age-standardized incidence rates (ASIRs), age-standardized death rates (ASDRs), and DALYs for GBTCs. These metrics characterized the burden across regions and nations from 1990 to 2021. Temporal trends were quantified using estimated annual percentage changes (EAPCs). Positive EAPC values with 95% CI excluding zero indicated increasing trends, while negative values denoted decreasing trends. This approach provided a consolidated overview of mortality and DALY rate trajectories over the three-decade study period.

### Decomposition analysis

Das Gupta decomposition analysis was employed to quantify the relative contributions of demographic and epidemiological factors – specifically age structure, temporal shifts in epidemiological rates, and population dynamics – to observed temporal changes in deaths and DALYs attributable to GBTCs. This method partitions the overall change into three components: (1) epidemiological changes (reflecting shifts in age-specific disease risk), (2) population growth, and (3) population aging. By isolating these constituent drivers, the analysis elucidates the principal mechanisms underpinning longitudinal transitions in GBTC burden and enables discrimination between changes driven by alterations in true disease risk and those arising from demographic shifts. This approach provides critical insights for public health policy, as it delineates whether observed increases (or decreases) in cancer burden stem primarily from modifiable risk factors or immutable demographic forces.

### Cross-country inequality analysis

To quantify cross-national inequalities in GBTC burden, two validated metrics were employed: the slope index of inequality (SII) and the concentration index^[^18^]^. The SII, derived through regression analysis, quantifies absolute health disparities by scaling country-level DALY rates against an SDI-based socioeconomic hierarchy. Higher SII values denote pronounced inequality in disease burden distribution. Complementarily, the concentration index – a relative measure grounded in the Lorenz concentration curve – assesses the extent to which health indicators are concentrated among specific socioeconomic strata. Negative concentration index values indicate disproportionate burden concentration among disadvantaged populations, whereas positive values signify concentration within advantaged groups.

### Predictive analysis

Projections of the global GBTC burden through 2050 were generated using a Bayesian age–period–cohort (BAPC) model with Integrated Nested Laplace Approximation (INLA). This methodology demonstrated superior predictive performance in comparative assessments relative to alternative projection approaches. The BAPC-INLA framework was specifically employed to forecast non-intervention GBTC mortality trajectories from 2022 to 2050, robustly accounting for age-stratified incidence and mortality patterns. Its computational architecture is uniquely suited for modeling future burden trends amid major demographic shifts (e.g., aging populations), providing policy-relevant time horizons for strategic health planning.

### MR

Causal effect estimation of body weight on cholangiocarcinoma (CCA) risk was performed using a two-sample MR framework, predicated on three core assumptions: (1) strong association between genetic variation and exposure; (2) independence of instruments from confounding factors; and (3) instruments affect outcome solely through exposure. Genetic instruments variables for body weight were derived from a publicly available genome-wide association study (GWAS) of 360 116 individuals. Single-nucleotide polymorphisms (SNPs) attaining genome-wide significance (P < 5 × 10^−8^) were selected, with clumping to ensure independence (linkage disequilibrium threshold: *R*^2^ < 0.001 within 10 000 kb), yielding 397 independent instrumental variables.

Summary statistics for CCA associations were obtained from the UK Biobank GWAS database, with no sample overlap with the exposure dataset and predominantly European ancestry. Instrument strength was quantified via F-statistics, with SNPs exhibiting *F* < 10 excluded to mitigate weak instrument bias. The mean *F*-statistic across all 397 instrumental SNPs was 41.7 (range: 10.2–157.3), substantially exceeding the conventional threshold of 10, thereby effectively minimizing potential weak instrument bias in our causal estimates.

Two-sample MR analyses were performed using R software (version 4.2.2), two-sample MR (version 0.5.6), and the MR-PRESSO package (version 1.0.0). We used several methods based on different hypotheses to derive potential causal relationships between exposures and outcomes: the inverse variance weighting (IVW) method, the MR-Egger method, the weighted median method (WME), and the Mendelian Randomization Pleiotropy RESidual Sum and Outlier (MR-PRESSO). The IVW method was used as the primary method to test causal relationships by performing a meta-analysis to test for causality. The IVW method was used as the primary method to test causality through meta-analysis of Wald associations for each instrumental variable (IV), WME, MR-Egger, and MR-PRESSO methods were used as Supplemental Digital Content Methods, available at: http://links.lww.com/JS9/H295 in this study. Heterogeneity was tested by Cochrane Q. *P* < 0.05 was considered significant heterogeneity, at which point a random-effects model was used for subsequent analyses; otherwise, a fixed-effects model was used. Stability of the MR results was determined by excluding instrumental variables one by one and using an omission sensitivity test. The effect of each included instrumental variable on causality was treated using the leave-one-out method and scatter plots were generated to visualize the results of the MR analyses.

Due to the limited availability of public GWAS data, the currently available and relatively sufficient statistical power pooled measure for CCA was used as the outcome for the two-sample MR To examine the causal relationship between BMI and CCA risk. The MR results directly support the causal association between BMI and the risk of CCA. Inferences about the overall burden of GBTC in a broader sense are only used as indicative evidence, and the boundaries and future research directions brought about by subtype heterogeneity and data availability are specifically discussed within the limitations.

## Results

### Global, regional, and national burden and trends of GBTC from 1990 to 2021

The global burden of GBTC continued to increase overall. In 2021, there were 314.47 thousand (95% UI: 265.61–353.97) prevalent GBTC cases worldwide, with a prevalence of 3.98 per 100 000 population (95% UI: 3.37–4.49). From 1990 to 2021, the global number of GBTC cases increased by 142% (Table 1).

**Table 1:** The number of prevalent cases and current rates of GBTC in 1990 and 2021, and the EAPC from 1990 to 2021.

	Prevalent cases			Prevalent rates		
Location	1990_thousands (95%UI)	2021_thousands (95%UI)	Percentage change	1990_per 100 000 (95%UI)	2021_per 100 000 (95%UI)	EAPC (95%UI)
Andean Latin America	0.98 (0.75–1.15)	2.53 (1.93–3.32)	1.58	2.59 (1.97–3.02)	3.83 (2.92–5.01)	1.24 (1.07–1.41)
Australasia	1.62 (1.53–1.7)	4.89 (4.34–5.27)	2.02	7.99 (7.56–8.39)	15.79 (14.03–17.04)	2.33 (1.99–2.67)
Caribbean	0.44 (0.39–0.48)	0.6 (0.53–0.69)	0.36	1.24 (1.1–1.36)	1.27 (1.11–1.45)	−0.11 (−0.23–0.01)
Central Asia	0.5 (0.44–0.58)	0.68 (0.6–0.77)	0.36	0.72 (0.64–0.84)	0.71 (0.63–0.8)	−0.59 (−1.11–0.06)
Central Europe	6.68 (6.26–6.97)	7.56 (6.83–8.3)	0.13	5.34 (5.01–5.57)	6.56 (5.93–7.2)	0.6 (0.57–0.63)
Central Latin America	3.49 (3.39–3.56)	5.9 (5.26–6.58)	0.69	2.12 (2.06–2.17)	2.33 (2.08–2.6)	−0.01 (−0.24–0.22)
Central Sub-Saharan Africa	0.06 (0.04–0.09)	0.17 (0.11–0.24)	1.83	0.12 (0.08–0.17)	0.12 (0.08–0.18)	0.26 (0.04–0.48)
East Asia	18.84 (14.41–23.66)	82.25 (55.95–106.88)	3.37	1.55 (1.18–1.94)	5.58 (3.8–7.26)	4.5 (4.31–4.7)
Eastern Europe	5.43 (5.09–5.8)	9.95 (9.21–10.7)	0.83	2.4 (2.25–2.56)	4.81 (4.45–5.18)	1.93 (1.59–2.27)
Eastern Sub-Saharan Africa	0.73 (0.49–0.99)	1.49 (1.05–1.98)	1.04	0.38 (0.25–0.52)	0.35 (0.25–0.47)	−0.45 (−0.67–0.23)
Global	130.03 (118.06–139.78)	314.47 (265.61–353.97)	1.42	2.44 (2.21–2.62)	3.98 (3.37–4.49)	1.63 (1.54–1.72)
High-income Asia Pacific	22.91 (21.22–24.12)	52.5 (44.3–59.12)	1.29	13.21 (12.24–13.91)	28.31 (23.89–31.88)	2.59 (2.49–2.7)
High-income North America	17.2 (16.12–17.82)	33.06 (30.26–34.71)	0.92	6.11 (5.73–6.33)	8.93 (8.17–9.38)	1.21 (1.14–1.28)
High-middle SDI	31.1 (27.27–33.31)	80.6 (60.62–93.66)	1.59	2.92 (2.56–3.13)	6.18 (4.65–7.18)	2.46 (2.33–2.58)
High SDI	66.95 (62.84–69.49)	133.65 (119.7–144.82)	1	7.61 (7.14–7.9)	12.22 (10.94–13.24)	1.58 (1.52–1.63)
Low-middle SDI	9.07 (7.7–12.77)	26.73 (21–33.09)	1.95	0.78 (0.66–1.1)	1.39 (1.09–1.72)	1.96 (1.89–2.03)
Low SDI	2.25 (1.76–3.11)	6.17 (4.28–7.73)	1.74	0.45 (0.35–0.62)	0.55 (0.38–0.69)	0.71 (0.61–0.81)
Middle SDI	20.5 (17.68–26.02)	67.12 (53.67–88.24)	2.27	1.19 (1.03–1.51)	2.74 (2.19–3.6)	2.75 (2.55–2.95)
North Africa and Middle East	2.28 (1.81–2.94)	6.94 (5.09–8.52)	2.04	0.67 (0.53–0.87)	1.11 (0.82–1.37)	1.82 (1.65–2)
Oceania	0.02 (0.01–0.02)	0.04 (0.03–0.05)	1	0.28 (0.16–0.38)	0.28 (0.19–0.37)	0.02 (−0.08–0.12)
South Asia	9.5 (7.63–13.62)	34.1 (23.67–40.37)	2.59	0.87 (0.7–1.25)	1.85 (1.28–2.19)	2.51 (2.42–2.6)
Southeast Asia	4.29 (3.07–5.36)	14.4 (10.12–18.34)	2.36	0.92 (0.66–1.15)	2.06 (1.45–2.63)	2.51 (2.4–2.62)
Southern Latin America	4.11 (3.94–4.27)	5.47 (5.07–5.81)	0.33	8.3 (7.96–8.63)	8.08 (7.49–8.58)	−0.19 (−0.26–0.11)
Southern Sub-Saharan Africa	0.18 (0.14–0.24)	0.48 (0.33–0.56)	1.67	0.35 (0.26–0.46)	0.59 (0.41–0.7)	1.97 (1.89–2.05)
Tropical Latin America	2.79 (2.66–2.89)	6.32 (5.89–6.61)	1.27	1.83 (1.75–1.89)	2.78 (2.59–2.9)	1.22 (1.03–1.41)
Western Europe	27.94 (26.32–29)	45.04 (40.33–48.22)	0.61	7.27 (6.85–7.54)	10.3 (9.22–11.02)	1.19 (1.09–1.29)
Western Sub-Saharan Africa	0.04 (0.03–0.05)	0.1 (0.06–0.12)	1.5	0.02 (0.02–0.03)	0.02 (0.01–0.02)	0.5 (0.12–0.88)

EAPC, estimated annual percentage change; SDI, socio-demographic index; UI, uncertainty interval

The global incident cases of GBTC were 216.77 thousand in 2021, with the incidence rate rising from 2.02 per 100 000 (95% UI: 1.82–2.2) in 1990 to 2.75 per 100 000 (95% UI: 2.3–3.11) in 2021, demonstrating an upward trend (EAPC 0.97, 95% UI: 0.88–1.06) (Supplemental Digital Content Table S1, available at: http://links.lww.com/JS9/H286). Compared with 1990, the number of deaths increased by 74%, from 98 680 to 171 960, and the global mortality rate per 100 000 population rose from 1.85 to 2.18 (EAPC 0.47) (Supplemental Digital Content Table S2, available at: http://links.lww.com/JS9/H287). DALYs increased by 60%, from 2.3261 million in 1990 to 3.7321 million in 2021 (EAPC 0.19) (Supplemental Digital Content Table S3, available at: http://links.lww.com/JS9/H288).

Significant disparities in GBTC burden were observed across global regions categorized by SDI. DALYs, mortality, incidence, and prevalence showed remarkable inequalities and upward trends across SDI groups. East Asia exhibited the highest increase in prevalent cases (3.37-fold) with an EAPC of 4.5% (95% UI: 4.31–4.70).

EAPC trends further highlighted regional differences: while high-SDI regions showed a negative EAPC (−0.6), all other SDI regions exhibited increasing GBTC burden, indicating substantial impacts of healthcare resource allocation and disease control efficiency. Since 1990, prevalence and incidence have risen in all regions, most notably in high-SDI regions, where mortality remained relatively stable. In contrast, low- and low-middle-SDI regions showed rising mortality and DALYs rates, suggesting ongoing aggravation of disease burden.

**Figure 1.**
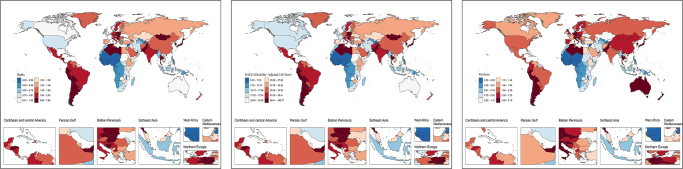
GBTC burden in 204 countries and territories worldwide in 2021. (a) ASMR. (b) ASDR. (c) ASIR. ASDR, age-standardized disability-adjusted life years rate; ASIR, age-standardized incidence rate; ASMR, age-standardized mortality rate; GBTC, gallbladder and biliary tract cancer.

Age-standardized mortality from GBTC peaks above 3.0 per 100 000 in the Andean nations of Chile, Bolivia and Peru, selected Eastern European states, and parts of South Asia (notably India, Bangladesh and Nepal), whereas rates remain below 0.6 per 100 000 across much of sub-Saharan Africa, northern Europe, and North America (Fig. 1). DALY burden parallels this pattern, exceeding 50 per 100 000 in the Andean region and South Asia, but falling beneath 15 per 100 000 in West Africa, Scandinavia, and Australasia. Similarly, age-standardized incidence surpasses 6.0 per 100 000 in Chile, Bolivia, Bangladesh, and Central Asia, yet remains under 1.4 per 100 000 in West African countries, North America, and northern Europe. Regional insets further highlight elevated GBTC burden in the Caribbean/Central America, moderate-to-high rates across the Persian Gulf and Eastern Mediterranean, marked heterogeneity on the Balkan Peninsula, high rates in Southeast Asia, and uniformly low burden in West Africa and northern Europe.

**Figure 2.**
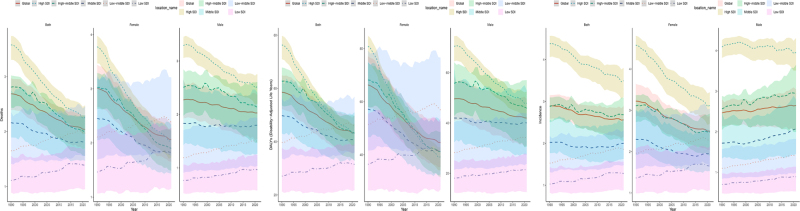
Temporal trends in age-standardized incidence, mortality, and DALYs of GBTC by gender across different SDI regions, 1990–2021. DALYs, disability-adjusted life years; GBTC, gallbladder and biliary tract cancer; SDI, socio-demographic index.

From 1990 to 2021, the global age-standardized incidence of GBTC remained largely stable, with the lowest rates observed in high-SDI regions and relatively higher rates in middle- and low-SDI areas (Fig. 2). While incidence rates in high-middle-SDI regions showed a slight decline over the period, rates in lower-SDI regions exhibited a more gradual increase. Gender-specific trends were similar, with male rates generally lower in high-SDI regions and converging with female rates in lower-SDI regions by 2021.

Age-standardized mortality also demonstrated a global decline, with the steepest reductions seen in high-SDI regions. In contrast, middle- and low-SDI regions experienced more moderate reductions, and low-SDI regions saw a slight rise in mortality. Mortality rates were consistently higher in males compared to females across all SDI groups.

DALY rates followed a similar trajectory, with the greatest reductions occurring in high-SDI regions. While high-middle and middle-SDI regions saw notable declines, low-middle-SDI regions showed smaller decreases, and low-SDI regions experienced a slight increase. Female DALYs decreased across all SDI groups, while male DALYs remained higher, especially in lower-SDI regions.

**Figure 3.**
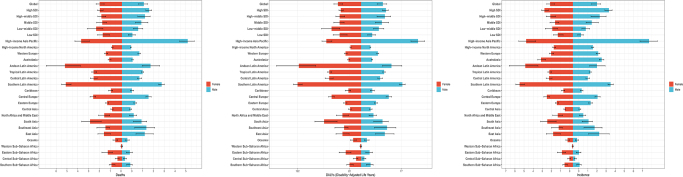
The age-specific rates for GBTC deaths, DALYs, and incidence, stratified by gender across different SDI regions in 2021. DALYs, disability-adjusted life years; GBTC, gallbladder and biliary tract cancer; SDI, socio-demographic index.

Substantial variations in the ASRs of GBTC-related deaths, incidence, and DALYs were observed across SDI regions and between sexes in 2021 (Fig. 3). Across the 21 GBD regions, high-income countries exhibited the highest ASIRs of GBTC, particularly among females, whereas low-SDI regions consistently showed the lowest rates across all three metrics in both sexes. South Asia accounted for a disproportionate share of age-standardized deaths and DALYs in both males and females despite moderate incidence rates. In contrast, Central and Eastern Europe showed elevated mortality and DALY rates, particularly among males. By sex, notable differences were evident across all metrics: females generally experienced higher incidence and DALY rates than males in most regions, particularly in high- and high-middle-SDI settings, whereas male mortality rates were higher than those of females in several regions, including Central Europe, Andean Latin America, and Eastern Europe.

### Decomposition analysis of GBTC burden by SDI

**Figure 4.**
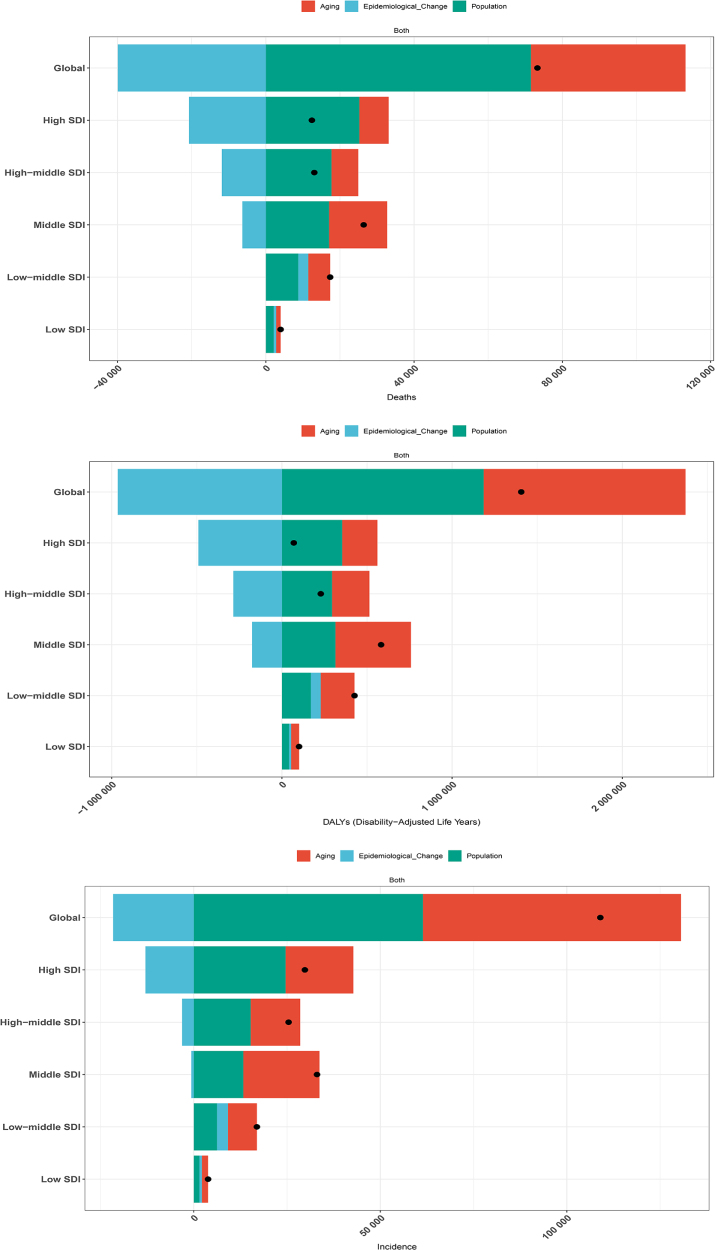
Decomposition analysis of changes in the deaths, DALYs, and incidence of GBTC from 1990 to 2021 across SDI regions. DALYs, disability-adjusted life years; GBTC, gallbladder and biliary tract cancer; SDI, socio-demographic index.

A decomposition analysis was conducted to evaluate the contributions of aging, population growth, and epidemiological changes to the burden of deaths, DALYs, and incidence of GBTC across SDI regions (Fig. 4). Globally, the increase in death rates was predominantly driven by population growth, with aging contributing to a lesser extent and epidemiological changes having minimal or negative effects. This pattern was consistent across SDI regions, with high-SDI regions showing the largest contributions from population growth and aging, while epidemiological changes had a more significant impact in low-middle and low-SDI regions.

For DALYs, population growth was the leading contributor globally and in high-SDI regions, with aging also playing a notable role. Conversely, in low-middle and low-SDI regions, epidemiological changes were more influential, reflecting shifts in disease patterns and risk factors. Similar trends were observed for incidence, where population growth and aging were key drivers in higher-SDI regions, while epidemiological changes had a more pronounced impact in lower-SDI regions.

These findings underscore that the rising global burden of GBTC is primarily driven by demographic changes, particularly population growth and aging, with regional differences in the influence of epidemiological factors.

### Global GBTC burden stratified by age and gender

**Figure 5.**
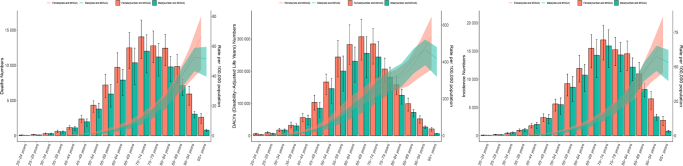
Age-standardized rates of GBTC mortality, incidence, DALYs, and age-specific DALYs rate per 100 000 Population by age group and gender in 2021. DALYs, disability-adjusted life years; GBTC, gallbladder and biliary tract cancer.

In 2021, the age-specific rates of death, DALYs, and incidence for GBTC showed a clear increasing trend with age, with mortality and DALYs peaking in the 90 + age group (Fig. 5). Notably, in both females and males, the burden increased significantly in older age cohorts, particularly from 65 to 69 years onward. However, the female mortality and DALY rates consistently outpaced those of males across all age groups. Female mortality rates were highest in the 85–89 age group, while the peak DALY rates occurred in the 80–84 age group. In contrast, male rates peaked slightly later, in the 90 + age group for mortality and in the 85–89 age group for DALYs. Incidence rates followed a similar pattern, with a marked increase in the elderly population, particularly in the 75–79 years and above age groups.

### Relationship between SDI and GBTC disease burden

**Figure 6.**
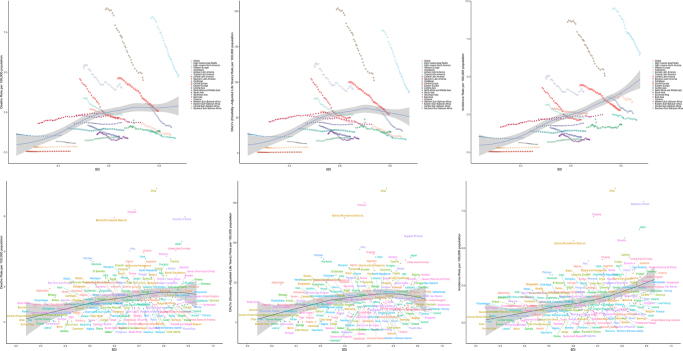
Age-standardized DALY rate, ASIR, and ASMR of GBTC in 21 GBD regions by the SDI, 1990–2021. ASIR, age-standardized incidence rate; ASMR, age-standardized mortality rate; GBTC, gallbladder and biliary tract cancer; SDI, socio-demographic index.

From 1990 to 2021, the burden of GBTC, measured by age-standardized mortality rate (ASMR), DALYs and incidence, showed a positive correlation with the SDI (Fig. 6). This relationship persisted until an SDI of approximately 0.7, beyond which both ASMR and ASDR began to decline. Notably, regions such as Andean Latin America, Central Europe, Southern Latin America, and high-income Asia-Pacific demonstrated ASMR and ASDR values that exceeded expectations for their SDI levels. At the national level, the correlation between GBTC burden and SDI reflected similar trends, with rates increasing up to an SDI of around 0.8, after which a decline was observed. The analysis further revealed significant negative correlations between EAPCs and ASMR/ASDR, with the Human Development Index in 2021 showing a strong negative correlation with EAPCs for both ASMR and ASDR.

### Cross-country inequality analysis of the burden of disease in GBTC

**Figure 7.**
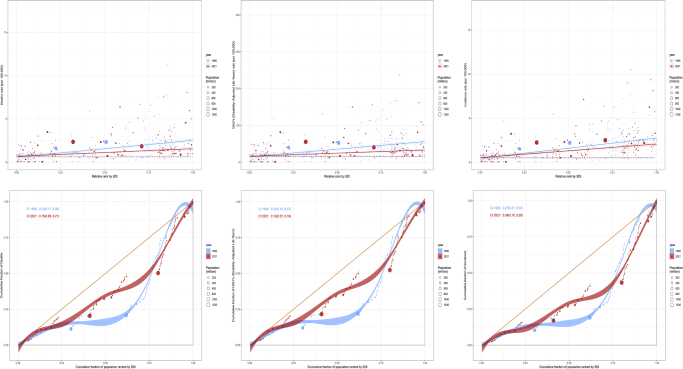
Inequality analysis of DALYs, ASIR, and ASMR in GBTC in 1990 and 2021 across the world. ASIR, age-standardized incidence rate; ASMR, age-standardized mortality rate; DALYs, disability-adjusted life years; GBTC, gallbladder and biliary tract cancer.

Inequality analysis of the global burden of GBTC in 1990 and 2021 revealed significant relative and absolute inequalities across SDI regions (Fig. 7). From 1990 to 2021, there was an increasing trend in SII, reflecting a growing disparity in GBTC burden between high and low-SDI regions. Higher burdens of GBTC were disproportionately concentrated in higher-SDI regions. The SII for DALYs increased from 7.16 in 1990 to 8.43 in 2021, indicating a widening gap in the DALY rates between the highest- and lowest-SDI regions. Similarly, concentration index for DALYs decreased from 0.48 in 1990 to 0.33 in 2021, demonstrating persistent inequalities, though the relative concentration of the burden in high-SDI regions diminished. For mortality, the SII increased from 0.31 in 1990 to 0.41 in 2021, while the CI for death rates decreased from 0.53 to 0.39 over the same period, further highlighting ongoing inequalities, though with a reduced concentration of mortality in higher-SDI regions. These findings underscore the persistent yet evolving inequalities in the burden of GBTC across SDI regions.

### Projection of GBTC burden up to 2050

**Figure 8.**
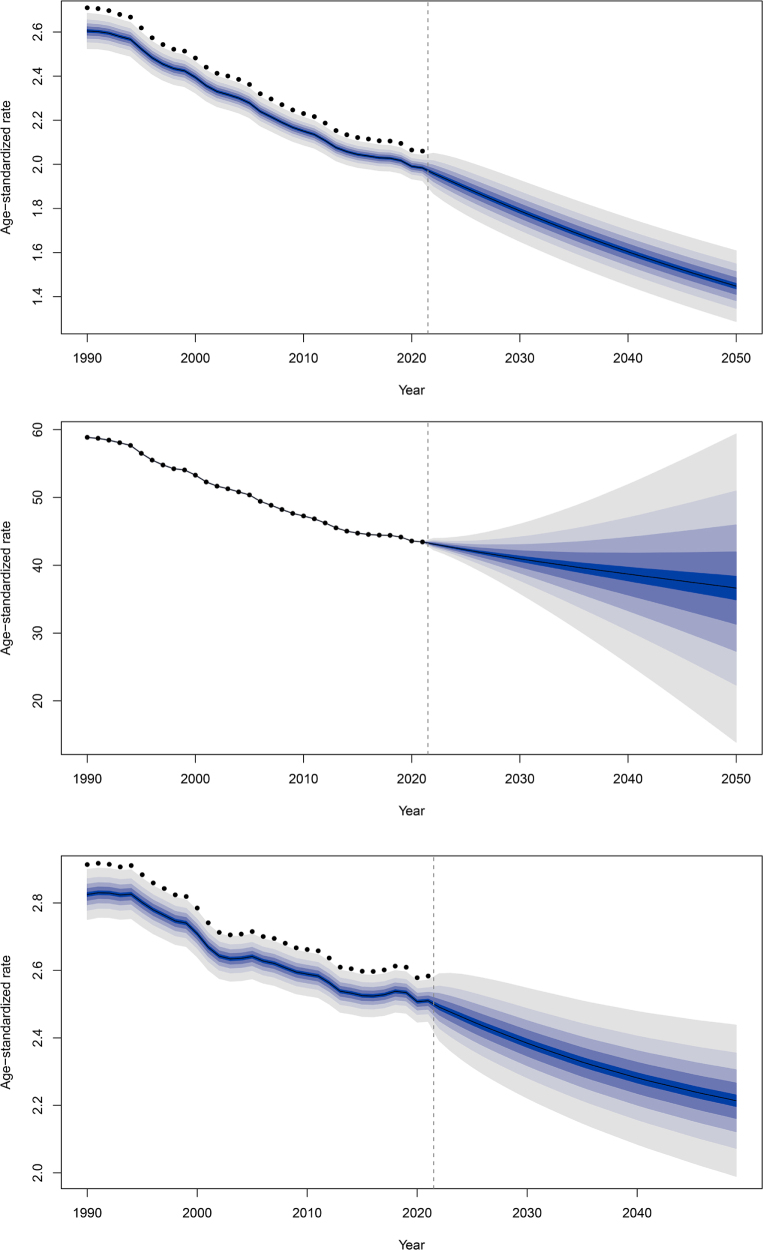
Projections for GBTC burden up to 2050. (a) Mortality, (b) DALYs, and (c) incidence. DALYs, disability-adjusted life years; GBTC, gallbladder and biliary tract cancer.

Under BAPC modeling, all three age-standardized metrics of GBTC – incidence, mortality, and DALYs – are projected to continue declining through 2050 (Fig. 8). The age-standardized mortality rate is forecast to maintain its downward trajectory post-2021, accompanied by widening UI. Age-standardized DALY rates likewise are expected to fall steadily over the projection period. Incidence rates are projected to decrease at a more gradual pace but nonetheless follow the same overall downward trend. Across all metrics, the expanding confidence envelopes beyond 2021 reflect growing projection uncertainty, yet consistently indicate a reduced global GBTC burden by mid-century.

Shaded areas represent 95% UI. The widening of intervals toward 2050 illustrates the growing uncertainty in forecasting distant future epidemiological rates, whereas the central projection trends (solid lines) indicate the modeled direction of change

### Risk factor for GBTC

**Figure 9.**
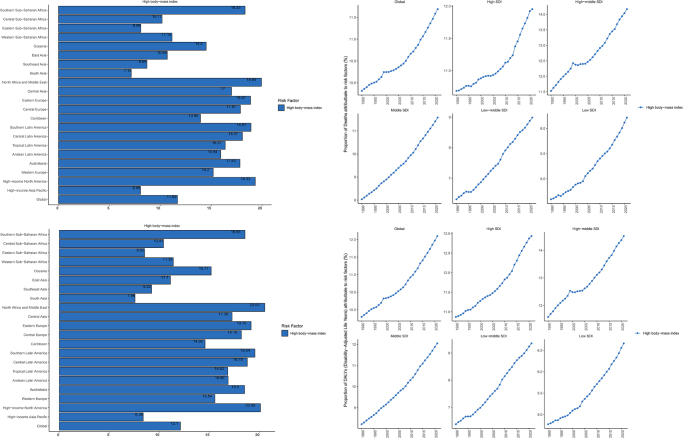
Proportion of GBTC burden attributable to high body mass index (BMI), stratified by SDI from 1990 to 2021. GBTC, gallbladder and biliary tract cancer; SDI, socio-demographic index.

From 1990 to 2021, the proportion of GBTC burden attributable to elevated BMI increased across all SDI quintiles (Fig. 9). High-SDI regions exhibited the steepest rise in BMI-attributable deaths and DALYs, whereas low-SDI settings showed a more gradual upward shift. Middle- and high-middle-SDI regions demonstrated intermediate trajectories. Throughout the period, the fraction of GBTC burden linked to high BMI was consistently greater in higher-SDI strata and remained lower in the least developed settings, reflecting a widening SDI-related gradient in metabolic risk contribution.

### MR analysis

Two-sample MR analysis was conducted to examine the causal association between body weight and CCA. A total of 397 SNPs associated with BMI were selected as instrumental variables (IVs), with all IVs demonstrating *F*-values >10, indicating that weak instrument bias did not substantially affect causal estimates (Supplemental Digital Content Table S4, available at: http://links.lww.com/JS9/H289). Cochran’s Q test showed no evidence of heterogeneity (Q = 360.0891, *P* = 0.7261), justifying the use of a fixed-effect model in the primary MR analysis. The inverse-variance weighted (IVW) method was employed as the primary analytical approach.

**Figure 10.**
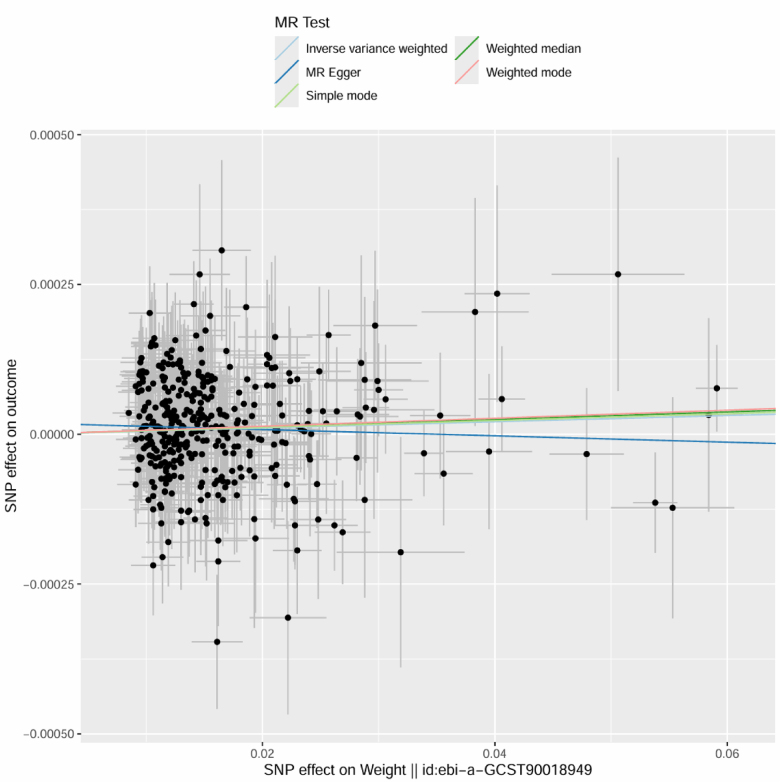
Scatter plot of body weight impact on cholangiocarcinoma.

**Figure 11.**
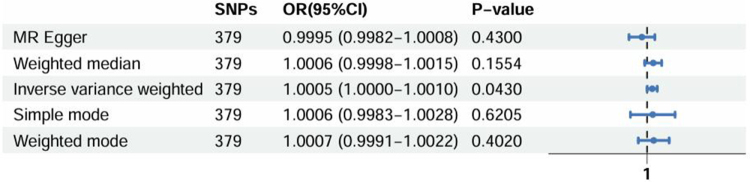
Results of two-sample MR analysis based on fixed-effects modeling. MR, Mendelian randomization.

IVW analysis revealed a significant causal association between body weight and CCA (odds ratio [OR] = 1.0005, 95% confidence interval [CI] [1.0000, 1.0010], *P* = 0.0430) (Figs 10 and 11, Supplemental Digital Content Figure S1, available at: http://links.lww.com/JS9/H285). Consistent results were observed with the weighted median estimator (OR = 1.0006, 95% CI: 0.9998–1.0015, *P* = 0.1554) and weighted mode (WM) method (OR = 1.0007, 95% CI: 0.9991–1.0022, *P* = 0.4020). Leave-one-out sensitivity analyses confirmed the robustness of MR inferences (Supplemental Digital Content Figure S2, available at: http://links.lww.com/JS9/H285). Tests for pleiotropy and heterogeneity showed no evidence of horizontal pleiotropy (*P* = 0.0899) or heterogeneity (*P* = 0.7017) in the weight–CCA risk association (Supplemental Digital Content Table S5, available at: http://links.lww.com/JS9/H290). Conversely, reverse-direction analysis treating CCA risk as the exposure revealed no statistically significant association with body weight (Supplemental Digital Content Figure S3, available at: http://links.lww.com/JS9/H285).

The solid line slope represents the association strength estimated by MR analysis, illustrating the genetically predicted BMI and its correlation with CCA risk.

## Discussion

Based on GBD 2021 data, we systematically analyzed the dynamic changes in global GBTC disease burden from 1990 to 2021. The global number of prevalent GBTC cases increased by 142%, deaths by 74%, and DALYs by 60%, clearly highlighting the continuous aggravation of this disease burden over the past three decades. In terms of ASRs, the EAPC was 0.97 for incidence (ASIR), 0.47 for mortality (ASMR), and 0.19 for DALYs rate (ASDR), indicating that although absolute case numbers increased significantly, the growth trends of ASRs were relatively moderate. This apparent paradox can be explained by the dual effects of demographic changes and medical advancements. On the one hand, global population growth and aging directly expanded the case base. Over time, the global population has continued to rise, and life expectancy has increased, leading to a growing proportion of older adults – who are at higher risk of GBTC – thereby substantially increasing absolute case numbers. On the other hand, improvements in diagnostic technologies and the implementation of prevention measures in some regions have partially mitigated the rise in ASRs. The widespread application of advanced imaging techniques (e.g., multi-slice spiral CT, magnetic resonance cholangiopancreatography) has improved early diagnostic rates, enabling more patients to be detected and treated at early stages, thus reducing disease progression and mortality rates and slowing the increase in ASRs.

Age-stratified analysis showed that GBTC mortality peaked in the 95+ years age group, with the highest DALY rate observed in the 90–94 years age group, reflecting the severe threat of the disease to older populations. With increasing age, various physiological functions decline, accompanied by reduced immune surveillance and clearance of tumor cells, thereby enhancing GBTC susceptibility and disease severity. Females exhibited higher death counts and DALYs across all age groups, with significantly higher DALY rates in 30–69-year-old women and the largest absolute DALY numbers in 45–74-year-old women. This gender disparity is intimately linked to biological factors: women have a twofold higher prevalence of cholelithiasis, and globally, women exhibit higher obesity prevalence than men^[^18,19^]^. Fluctuations in estrogen levels may alter bile composition and gallbladder contractility, promoting gallstone formation. Chronic inflammation induced by long-standing gallstones irritates the gallbladder and biliary mucosa, increasing GBTC risk. Additionally, pregnancy history may facilitate gallbladder cancer development by influencing bile composition; multiple pregnancies cause hormonal fluctuations that disrupt bile secretion and excretion, elevating the incidence of cholelithiasis and GBTC^[^20,21^]^.

SDI-stratified analysis unveiled a non-linear association between socioeconomic development and GBTC burden, where ASMR and ASDR first increased and then decreased with rising SDI, peaking at an SDI of approximately 0.7. Despite advanced medical technologies in high-SDI regions, ASIR and ASMR remained elevated, likely associated with the prevalence of modifiable risk factors such as high obesity rates and westernized dietary patterns^[^22–24^]^. In these regions, higher living standards are characterized by increased consumption of high-calorie, high-fat, and high-sugar diets, coupled with reduced physical activity, leading to widespread obesity^[^25^]^. Studies have shown a positive correlation between obesity and GBTC risk^[^10,13^]^, whereby hormonal imbalances and metabolic by-products in obese individuals may disrupt biliary physiology and promote tumorigenesis^[^26,27^]^.

Conversely, low-middle-SDI regions exhibited the highest ASDR, reflecting critical deficits in late-stage diagnosis and treatment accessibility. Limited economic development in these areas is often accompanied by inadequate healthcare infrastructure, scarce medical resources, and shortages of specialized healthcare personnel, resulting in delayed diagnosis – most patients present at advanced stages when symptoms become overt^[^28^]^. This leads to higher mortality and DALYs due to difficult treatment and poor prognosis. Notably, East Asia showed the steepest increase in prevalent cases with an EAPC of 4.5%, highlighting an urgent need for strengthened prevention systems. Rapid socioeconomic transition in East Asia has triggered profound lifestyle and dietary shifts, contributing to rising incidences of metabolic diseases (e.g., obesity, diabetes), which may drive the GBTC burden surge^[^29^]^. Accelerated population aging further elevates GBTC risk in this region.

Epidemiological changes (i.e., changes in age-specific risk) reflects variations in age-specific rates/risks. In low-/lower-middle-SDI regions, its positive contribution may stem from the following non-mutually exclusive mechanisms: (1) True increasing incidence: ongoing epidemiological transitions, characterized by dietary shifts toward processed foods and rising rates of metabolic disorders, may be increasing the population-level risk for gallstone disease, a major precursor for GBTC. (2) Inadequate diagnostic and therapeutic capabilities: in these regions, persistent deficits in healthcare access, late-stage diagnosis, and limited availability of curative surgery or systemic therapy likely lead to limited opportunities for patients to improve their quality of life in real-world settings. (3) Evolving diagnostic artifact: improvements in diagnostic imaging access, though still inadequate, may lead to improved case ascertainment over time. This would artifactually inflate the measured “epidemiological” trend, particularly for incidence, as historically missed cases become recorded. Disentangling these contributions with existing aggregate data is challenging. However, the concurrent rise in both mortality and DALY rates in these regions, as shown in our results, suggests that factors negatively impacting survival are dominant drivers over purely artifactual case-finding. This underscores an urgent need for health systems strengthening focused on early diagnosis and treatment access, alongside primary prevention of metabolic risk factors.

Decomposition analysis further revealed that population growth was the primary driver of increasing GBTC burden across all SDI regions, surpassing the impacts of aging and epidemiological changes. In low-SDI regions, epidemiological shifts exhibited a positive effect on burden, indicating rising actual disease risk, whereas population aging showed a negative correlation with burden.

Analysis using the SII and concentration index revealed that global cross-country inequalities in GBTC burden have diminished from 1990 to 2021, although absolute disparities remain substantial. The decline in SII indicates a narrowing gap between high- and low-income nations, likely reflecting improvements in global health resource allocation and wider dissemination of GBTC prevention and control knowledge. Enhanced aid from international agencies and national governments has bolstered healthcare infrastructure, fostered specialist training, and promoted GBTC awareness and technical capacity in low-income settings, thereby partially closing the gap with wealthier countries.

The socioeconomic distribution of GBTC risk factors further complicates global control efforts. In high-SDI regions, obesity and related metabolic risks are predominant drivers. Our analysis shows these regions not only exhibit the highest current fraction of BMI-attributable burden but also a steeply rising trajectory. Coupled with demographic projections indicating sustained growth in absolute case numbers due to population aging, these findings underscore a critical public health imperative. From an intervention perspective, our MR results, which provide genetic evidence for the causality of BMI, reinforce the biological plausibility and potential effectiveness of targeting this risk factor. Consequently, population-level strategies centered on weight management and metabolic risk control should be prioritized in high-SDI settings. Such strategies could include promoting healthy diets, physical activity, and systemic policies to curb obesity. We emphasize that while MR supports the feasibility of BMI-focused interventions, the precise quantitative impact of such measures on future burden reduction requires further prospective and modeling studies. In high-SDI regions, obesity and related metabolic risks can be mitigated through structured health-management programs, whereas in low-SDI settings, traditional drivers such as cholelithiasis remain poorly addressed owing to healthcare infrastructure constraints. Bridging this divide will require coordinated international action: enhancement of primary care capacity in resource-limited regions; deployment of affordable, user-friendly screening modalities (for example, portable ultrasonography); and strengthened global collaboration on risk-factor mitigation. Multilateral agencies should expand financial and technical assistance to bolster healthcare facilities and workforce training in low-SDI countries. Concurrently, nations must share best practices in GBTC prevention, jointly develop comprehensive control frameworks, and priorities equitable access to early detection and intervention, thereby advancing global health equity. Conversely, concentration index analysis underscores a persisting concentration of disease burden among vulnerable populations, notably in South Asia and southern Latin America. In these regions, poverty and limited education contribute to poor health literacy and hinder access to quality care. Uneven distribution of health services further marginalizes residents of remote and impoverished communities, while adverse living conditions and dietary patterns elevate exposure to GBTC risk factors, exacerbating the burden borne by these disadvantaged groups.

The BAPC model projections suggest a slow downward trend in global age-specified rates (ASIR, ASMR, ASDR) from 2021 to 2050 if current intervention levels are maintained. However, it is important to note that the absolute number of cases is driven by population growth (projected to reach 9.8 billion globally in 2050) and aging (over 22% of the population over 65 years of age), and the actual number of cases may increase by approximately 30%. Especially in low-SDI regions, the burden may continue to climb in absolute terms as the population base expands, despite declining standardized rates.

The progressive widening of UI in our BAPC projections toward 2050 (Fig. 8) reflects the inherent challenge of long-term forecasting, where uncertainties from demographic shifts, future risk factor trends, and potential healthcare interventions compound over time. This increased uncertainty primarily affects the precision of the predicted ASRs in the distant future. However, the directional trends – specifically the continued decline in age-standardized incidence, mortality, and DALY rates – remained robust across all model iterations. More importantly, the projection of a substantial rise in absolute case numbers (+30% by 2050 compared to 2021) demonstrates greater robustness. This finding is predominantly driven by deterministic demographic forces – namely, United Nation-projected population growth and aging – which are modeled with higher certainty than future epidemiological trends. Sensitivity analyses, in which we held age-specific rates constant at 2021 levels, still yielded an absolute case increase of approximately +22% by 2050 purely from demographic change. Therefore, while the exact magnitude of future ASRs is subject to uncertainty, the conclusion that the absolute GBTC burden will grow substantially due to demographic pressures is well founded and highlights an unavoidable public health challenge.

We conducted a two-sample-MR analysis to interrogate the causal effect of BMI on GBTC risk. Leveraging 397 BMI-associated independent SNPs as instrumental variables, IVW estimates indicated that each one-unit increase in BMI was associated with a modest but statistically significant elevation in GBTC risk (OR 1.0005; 95% CI: 1.0000–1.0010; *P* = 0.043). This genetic evidence supports a causal link between elevated BMI and increased risk of biliary tract cancer. Crucially, the MR framework moves beyond observational correlation to provide evidence that is less susceptible to confounding and reverse causation. Therefore, our findings lend causal-level support to the hypothesis that population-wide interventions aimed at reducing BMI could contribute to a future reduction in the burden of GBTC. Concordant findings from weighted median and WM methods, together with leave-one-out sensitivity analyses, affirmed the robustness of this association.

The population attributable fraction (PAG) is a function of both the relative risk and the prevalence of the exposure. Although the per-unit effect of the OR of MR is small, in populations with widespread high BMI exposure, the PAF is jointly determined by the magnitude of the effect and the prevalence of exposure. Consequently, it may correspond to a substantial population attributable burden. In high-SDI regions where overweight and obesity prevalence often exceeds 60%, even a small relative risk applied across a vast exposed population generates a substantial attributable burden, as reflected in our GBD-based PAF estimates. It underscores that the population burden of GBTC attributable to high BMI arises not from a large risk in a few individuals, but from a pervasive risk affecting the majority of adults in many societies, reinforcing the imperative for population-wide preventive strategies.

In this study, the MR analysis provided the causal direction and average effect estimate (reported as OR with 95% CI) between BMI and the outcome risk. In contrast, the PAF derived from the GBD study estimated the proportion of burden attributable to BMI under a theoretical minimum-risk exposure level, based on the population distribution of BMI exposure and its corresponding risk function (reported with 95% UI). Consequently, even a modest average causal effect per unit BMI may translate into a substantial and prominent population-attributable burden in populations where high BMI exposure is highly prevalent.

Given that high BMI is a key contributor to both gallstone disease (a major risk for gallbladder cancer) and metabolic dysfunction-associated fatty liver disease (a risk factor for intrahepatic CCA), this causal inference for CCA serves as strong indicative evidence for the “intervenability” of high BMI within the broader spectrum of GBTC. Future studies with subtype-stratified genetic data are needed to precisely quantify the causal effect of BMI on gallbladder cancer and different CCA subtypes.

The two-sample MR analysis utilized GWAS summary statistics for CCA, as this was the most specific GBTC subtype with publicly available, sufficiently powered genetic data. While this directly tests causality for CCA, the shared anatomical origin, pathophysiological pathways (e.g., inflammation, biliary stasis), and established epidemiological overlap with gallbladder cancer support the biological plausibility of extending the inferred causal relationship to the broader GBTC spectrum.

Complementary GBD data corroborate high BMI as a major contributor to GBTC burden, with pronounced regional and sex differences. In 2021, age-standardized mortality fractions attributable to elevated BMI peaked in North Africa and the Middle East, whereas South Asia exhibited the lowest BMI-attributable proportions. Globally, females demonstrated higher BMI-attributable death fractions than males, consistent with estrogen-mediated metabolic interactions. These findings underscore the need for targeted, region- and sex-specific interventions – ranging from lifestyle modification to early screening – to mitigate the GBTC burden attributable to adiposity^[^30–32^]^.

Recent epidemiological studies have established distinct risk factor profiles for gallbladder cancer (GBC) and CCA^[^33^]^. GBC risk is predominantly driven by gallstone disease, with a growing contribution from metabolic factors such as obesity and diabetes^[^34^]^. In contrast, CCA – particularly intrahepatic CCA (iCCA) – is strongly linked to chronic liver diseases^[^35^]^, including viral hepatitis, metabolic dysfunction-associated fatty liver disease (NAFLD)^[^36^]^, and cirrhosis, reflecting a “liver-origin” cancer pattern, while extrahepatic CCA (eCCA) is more closely associated with primary sclerosing cholangitis and gallstones.

Our burden estimates are derived from the GBD’s aggregate “GBTC” category, while our MR analysis specifically tested CCA risk. This discrepancy arises from data availability constraints: the GBD provides robust, comparable global estimates for the combined category, whereas large-scale genetic data are currently only accessible for the more specifically defined CCA. We acknowledge that etiological factors may vary between gallbladder cancer and CCA.

However, high BMI is a well-established risk factor for both subtypes through overlapping mechanisms involving chronic inflammation, metabolic hormone dysregulation, and gallstone disease. Therefore, while our MR estimates directly support a causal role for high BMI in CCA, we posit that the conclusion is strongly indicative of a broader relationship with GBTC. Future studies with subtype-stratified burden estimates and genetic data will be valuable to confirm and quantify potential differences.

The validity of our findings is contingent upon the completeness and accuracy of underlying data sources; in several low-SDI countries, cancer registry deficiencies may have led to missing or imprecise estimates. The absence of detailed anatomical subsite stratification precluded differentiation between gallbladder carcinoma and CCA by location. The MR analysis was conducted using genetic summary statistics for CCA. While this provides direct causal evidence for BMI’s role in CCA, and the biological rationale supports extending this inference to the broader GBTC category (as high BMI contributes to shared risk pathways like gallstones and metabolic syndrome), the estimated causal effect (OR) is specifically for the CCA outcome. The lack of subtype-specific genetic data for gallbladder cancer limits our ability to delineate precise causal estimates for each GBTC component. Future investigations should disaggregate GBTC subtypes, incorporate a broader spectrum of risk factors, and integrate molecular epidemiology and genomics to elucidate pathogenesis. Furthermore, GBD risk-factor assessment was limited to elevated BMI, omitting key contributors such as cholelithiasis and metabolic risk factors^[^37^]^, which may have resulted in underestimation of attributable burden. Future investigations should disaggregate GBTC subtypes, incorporate a broader spectrum of risk factors, and integrate molecular epidemiology and genomics to elucidate pathogenesis. Cost-effectiveness analyses are also warranted to inform prioritization of targeted prevention and control strategies.

## Data Availability

The datasets generated during and/or analyzed during the current study are available in the GBD repository (http://ghdx.healthdata.org/gbd-results-tool), and the UK Biobank GWAS database (https://gwas.mrcieu.ac.uk).

## Research registration unique identifying number (UIN)

No human subjects involved in this study.
